# Percutaneous Management of Hepatic Duct Injury Using Extra-Anatomic Biliary Catheters

**DOI:** 10.7759/cureus.35012

**Published:** 2023-02-15

**Authors:** Almamoon Justaniah, Mohamed Z Abughararah, Niaz Ahmad, Majed Ashour, Hassan Alqarni

**Affiliations:** 1 Department of Radiology, King Faisal Specialist Hospital and Research Centre, Jeddah, SAU; 2 General Surgery, Hepatobiliary Unit, King Faisal Specialist Hospital and Research Centre, Jeddah, SAU; 3 Surgery, St. James's University Hospital, Leeds, GBR

**Keywords:** catheters, extra-anatomic, hepatic duct injury, percutaneous, stent, extra anatomic, biliary injury, interventional radiology, bile duct

## Abstract

Iatrogenic bile duct injury during laparoscopic cholecystectomy is a known complication of low incidence. The outcome can be devastating if not recognized and managed timely and properly. In cases of iatrogenic biliary injury due to cholecystectomy, the management depends on the level of injury, the timing of discovery (intraoperative or postoperative), and the patient's condition. If discovered intraoperatively, the injury should be managed immediately. In case expertise is lacking, a surgical drain with external biliary drainage can provide a temporary alternative solution to allow for referral to a tertiary care center. If the patient is septic or not fit for surgery, a percutaneous internal-external biliary drainage (PTBD) catheter can be placed until the patient’s condition improves. We report a case of complete transection of the common hepatic duct during laparoscopic cholecystectomy managed by extra-anatomic PTBD.

## Introduction

Iatrogenic biliary injury is a recognized complication of cholecystectomy surgery [1], with potentially serious long-term consequences [2]. Whilst earlier studies reported the incidence of biliary injury at 0.6% for laparoscopic compared to 0.1% for open cholecystectomy [3], the more recent studies reported an overall incidence of 0.02%, particularly in high-volume centers [4].

Several strategies during laparoscopic cholecystectomy may help to reduce the incidence of biliary injury, including meticulous dissection of a distorted Calot's triangle, careful usage of the diathermy away from the bile ducts, and avoiding excessive traction on the gallbladder [5]. Better communication between the lead surgeon and the assisting surgeon during surgery and confirmation of the anatomy before dividing the cystic duct and cystic artery has also been shown to reduce the incidence of biliary injury [6]. 

When an inadvertent biliary injury is recognized during the surgery, corrective surgery (either by hepaticojejunostomy surgery or other surgical intervention depending on the findings) can be undertaken during the same surgery with a good outcome. However, if the injury is not recognized intraoperatively, the management depends on the time since the surgery, the presence of sepsis, and the patient’s condition. In the absence of sepsis, corrective surgery is to be performed within 72 hours. After 72 hours, or in the presence of sepsis or other complications, the surgery can be deferred and sepsis management with the insertion of a percutaneous internal-external biliary drainage (PTBD) and collection drainage should be undertaken. 

## Case presentation

**Figure 1:**
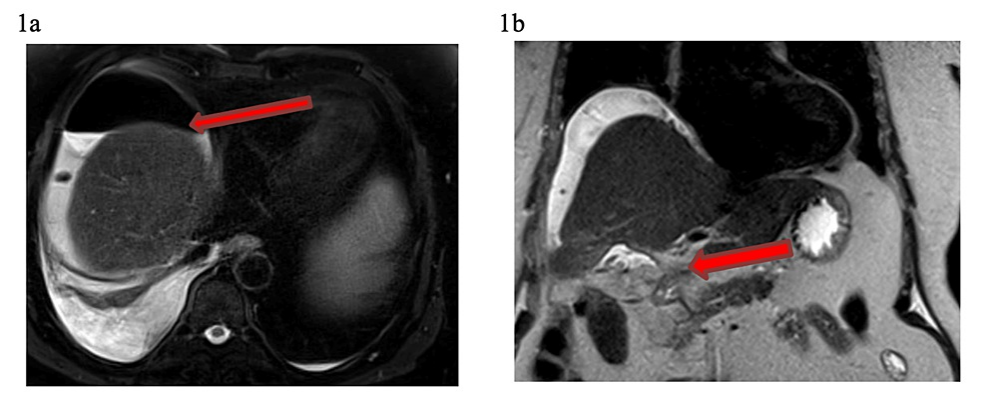
MRI without contrast. T2 fat saturation axial image demonstrates large perihepatic collection (1a). T2 coronal image demonstrated a small porta hepatis collection (1b). No intrahepatic biliary dilatation

An 85-year-old male with hypertension underwent laparoscopic cholecystectomy for biliary pancreatitis. During surgery, they converted from laparoscopic to open approach, due to the inflammation and omental adhesions. Bile leak was identified from the common hepatic duct (CHD), which was completely transected at the confluence (Bismuth type E4). Two 6 Fr. feeding tubes were inserted in each hepatic duct and externalized. A Jackson Pratt drain was placed in the subhepatic space and the patient was transferred to our institution seven days after the surgery (as he required tertiary center care). Upon admission, he was dehydrated, having pneumonia and acute kidney injury (not fit for surgical intervention). The magnetic resonance cholangiopancreatography (MRCP) demonstrated a complete transection of the CHD at the level of the hilum with biliary leak, perihepatic collection, porta-hepatis collection (biloma) (Figures 1A, 1B) as well as non-dilated biliary ducts.

**Figure 2:**
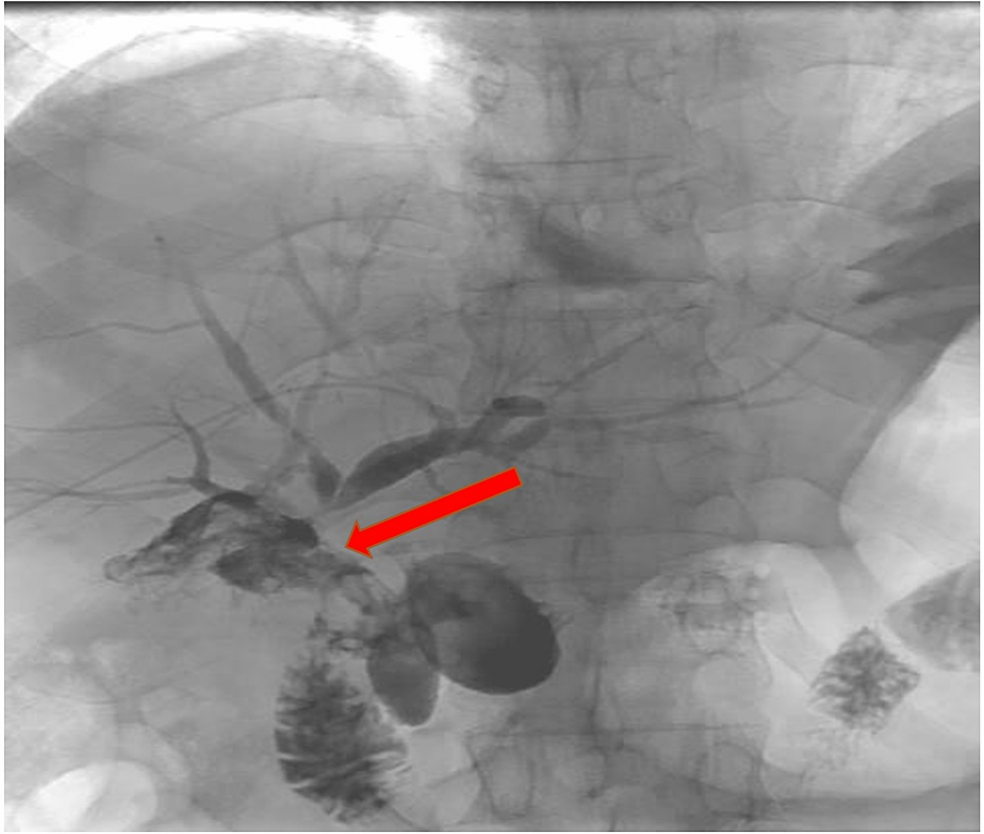
Percutaneous transhepatic cholangiogram (PTC). Left peripheral hepatics duct access from a right intercostal approach, demonstrating non-dilated ducts with contrast leak into the porta hepatis collection

A percutaneous transhepatic cholangiogram (PTC) was performed under general anesthesia confirming the CHD transection at the confluence with contrast leak into porta-hepatis (Figure 2).

**Figure 3:**
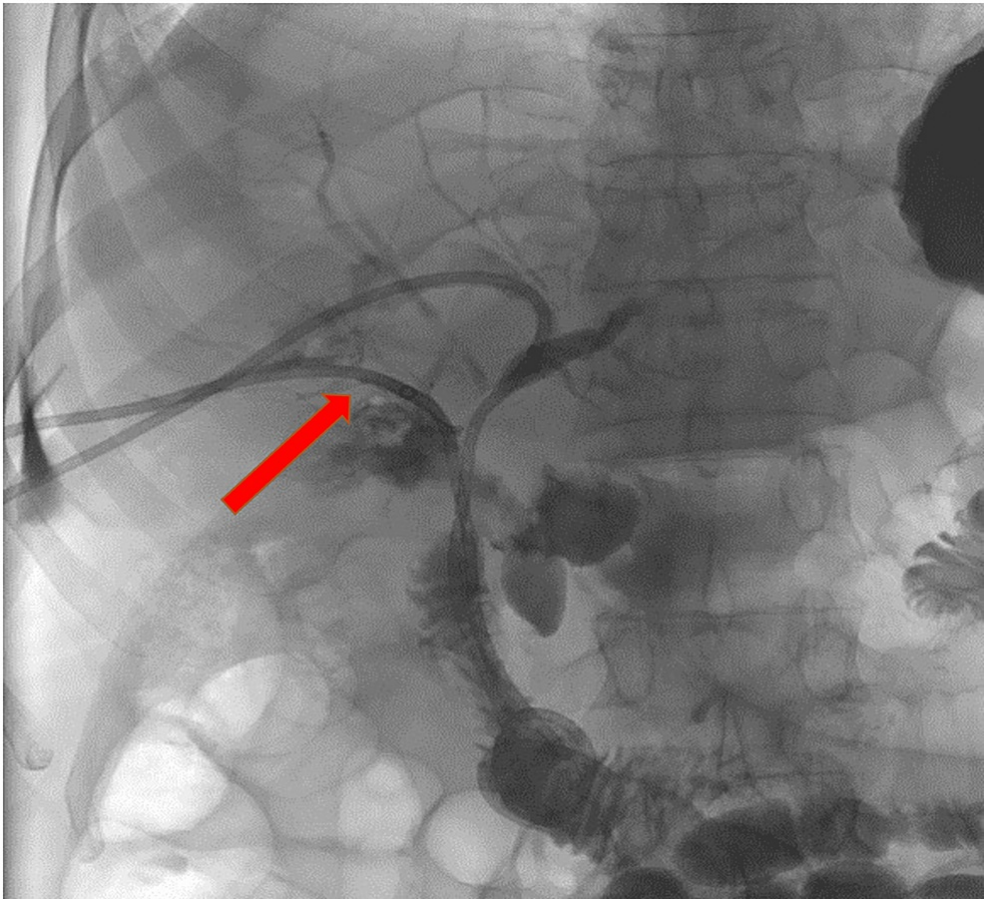
Percutaneous transhepatic cholangiogram (PTC) demonstrates two percutaneous internal-external biliary drainage (PTBD) catheters from a right intercostal approach draining the left and the right ducts separately, and passing to the duodenum via an extra-anatomic course

The left and right hepatic ducts were accessed separately from a right intercostal approach. Given the complete transection of the CHD, the wire inadvertently passed to the second part of the duodenum extraluminally/extra-anatomically, through a false tract from the porta-hepatic collection to the duodenum. Two internal-external 8.5 Fr. PTBDs were placed (Figure 3) and connected to drainage bags for one week, then capped.

The collection dried out and the drainage catheter was removed after two weeks. The patient was discharged home and the PTBDs were exchanged after three months. The patient continued to be unfit for surgery due to multiple comorbidities including a new stroke. 

**Figure 4:**
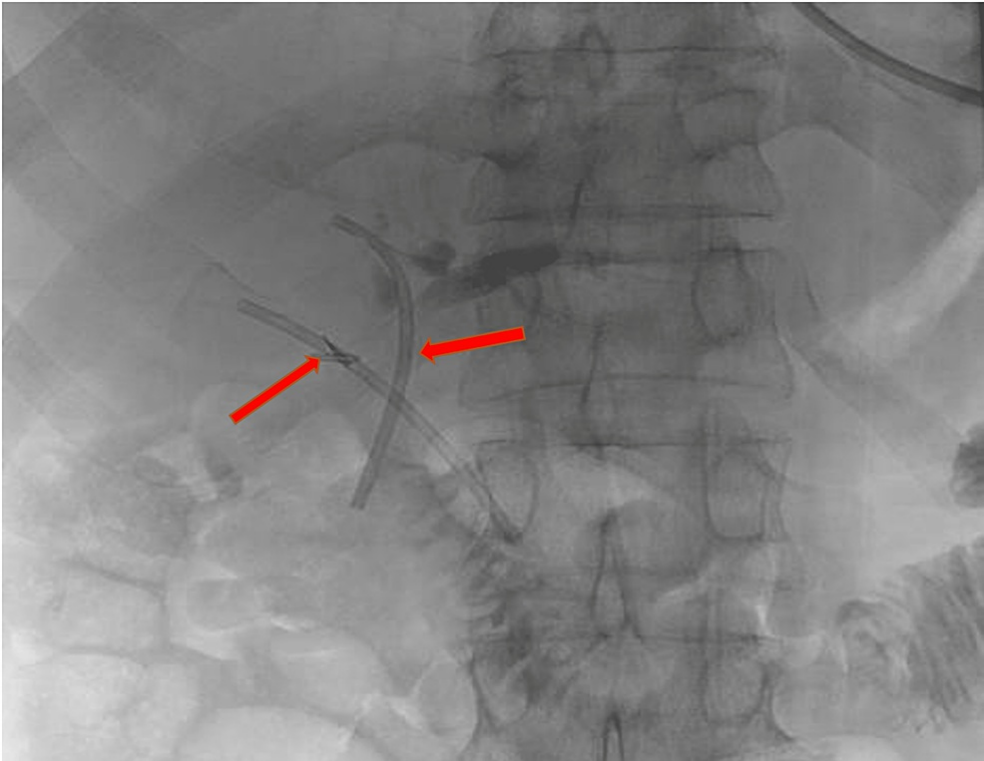
Placement of bilateral endoscopic plastic biliary stents percutaneously via the previous percutaneous internal-external biliary drainage (PTBD) accesses through extra-anatomical tracts to duodenum

At five months, a cholangiogram was performed demonstrating patent mature extra-anatomic tracts with no evidence of leak. Therefore, both PTBDs were removed and two 7 Fr. plastic endoscopic biliary stents were placed percutaneously (Figure 4), to be exchanged endoscopically.

**Figure 5:**
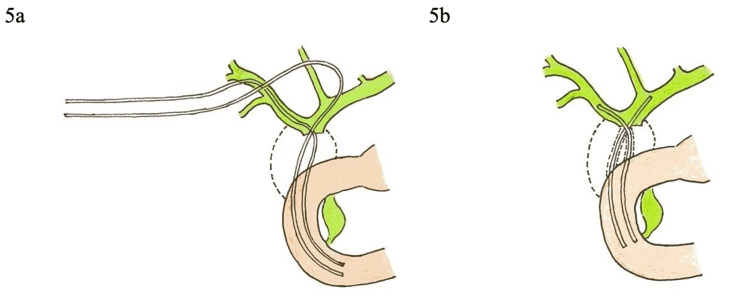
Illustrations demonstrate (a) Bilateral percutaneous internal-external biliary drainage (PTBD) via a right intercostal approach passing extra-anatomically to the duodenum (b) Five months later, two internal biliary plastic stents were placed percutaneously through the mature extra-anatomic tracts into the duodenum.

At one year, the biliary stents were exchanged endoscopically and the patient's overall condition was improving with no complications. Figures 5A, 5B illustrate the procedure of PTBD and the biliary stenting. 

## Discussion

Management of patients following major bile duct injury poses a challenge and requires experienced hepatobiliary surgeons. Early recognition is important to prevent major morbidity. An atypical postoperative course with pain, sepsis, jaundice, or bile-stained dressing should raise the suspicion of leak. Elevated bilirubin and alkaline phosphatase may suggest ligation or clipping of the bile duct; while bile leak from the wound may suggest transection of the bile duct, or leak from the gall bladder bed due to transection of the duct of Luschka. Ultrasound, computed tomography, and MRCP may detect the collection and/or the biliary dilation. Our patient presented with pneumonia and comorbidities. Hence, we proceeded with the nonsurgical option. While placing the PTBDs intraluminally is ideal for biliary diversion and healing of the injury, the complete CHD transection forced us to go extra-anatomically. Despite that, the patient did well for one year with further stent internalization and endoscopic exchange. Eikermann et al. described that in some cases, the nonsurgical intervention can provide a definitive treatment option [7]. 

Extra-anatomic biliary drainage and stenting have been described for biliary drainage in the setting of palliation for inoperable cholangiocarcinoma, anastomotic recurrence of the periampullary lesion, and biliary trauma. These included trans-tumoral, trans-parenchymal, and hepato-gastrostomy [8]. Percutaneous dilatation of spontaneous hepatoduodenostomy and stenting has also been described for malignant tumors and occasionally for benign diseases or anatomic disruptions [9-11]. Extra-anatomic biliary stenting in the setting of iatrogenic biliary injury has not been previously described. 

## Conclusions

Iatrogenic biliary injury during laparoscopic cholecystectomy is a rare complication and may have life-changing consequences without appropriate treatment. Recognition and timely treatment are critical. Surgical management should be carried out within 72 hours by a surgeon experienced in the procedure. Delayed surgical intervention is needed in patients where the biliary injury was unrecognized or immediate treatment was not possible. Extra-anatomic biliary catheters (EABS) offer a long-term alternative to surgical management and have some tangible benefits over hepaticojejunostomy in patients where surgery has concomitant elevated risk due to their poor performance status or comorbidities.
